# Feature Selection for Topological Proximity Prediction of Single-Cell Transcriptomic Profiles in *Drosophila* Embryo Using Genetic Algorithm

**DOI:** 10.3390/genes12010028

**Published:** 2020-12-28

**Authors:** Shruti Gupta, Ajay Kumar Verma, Shandar Ahmad

**Affiliations:** School of Computational and Integrative Sciences, Jawaharlal Nehru University, New Mehrauli Road, New Delhi 110067, India; shrutigupta217@gmail.com (S.G.); ajayverma81@gmail.com (A.K.V.)

**Keywords:** spatial organization, single-cell RNA sequencing, gene expression pattern, *Drosophila* embryo, DREAM challenge, genetic algorithm

## Abstract

Single-cell transcriptomics data, when combined with in situ hybridization patterns of specific genes, can help in recovering the spatial information lost during cell isolation. Dialogue for Reverse Engineering Assessments and Methods (DREAM) consortium conducted a crowd-sourced competition known as DREAM Single Cell Transcriptomics Challenge (SCTC) to predict the masked locations of single cells from a set of 60, 40 and 20 genes out of 84 in situ gene patterns known in *Drosophila* embryo. We applied a genetic algorithm (GA) to predict the most important genes that carry positional and proximity information of the single-cell origins, in combination with the base distance mapping algorithm DistMap. Resulting gene selection was found to perform well and was ranked among top 10 in two of the three sub-challenges. However, the details of the method did not make it to the main challenge publication, due to an intricate aggregation ranking. In this work, we discuss the detailed implementation of GA and its post-challenge parameterization, with a view to identify potential areas where GA-based approaches of gene-set selection for topological association prediction may be improved, to be more effective. We believe this work provides additional insights into the feature-selection strategies and their relevance to single-cell similarity prediction and will form a strong addendum to the recently published work from the consortium.

## 1. Introduction

The advancement in next-generation sequencing (NGS) methods, coupled with cell sorting and culturing, have made it possible to study the precise transcriptomic profiles of individual cells. The first account of single cells’ expression profiles captured by using NGS, in 2009, was a significant advancement over traditional bulk expression analysis [1]. RNA samples collected from bulk cells provided only the average gene expression value of an ensemble of single cells and hence failed to present the temporal and spatial variability across multiple similar groups of cells. Single-cell RNA sequencing (scRNAseq) has helped in unveiling new cell types and subpopulations of cells which were hitherto unknown [2,3]. This technology has been able to provide novel insights into the cellular compositions and biological processes, including dynamic processes involved in development and differentiation. Moreover, scRNAseq studies have also helped in uncovering the cellular heterogeneity in complex tissues and even in populations of seemingly similar cells. Spatial heterogeneity and mapping are now widely studied issues in the field of scRNAseq data analysis.

Through technological and computational advances, it has been realized that one of the major limitations of scRNAseq is the loss of spatial information during cell isolation; hence, much effort has been made to overcome this limitation, both in terms of experimental technology [4,5,6,7] and computational data analytics [8,9,10,11,12,13,14]. With the advancement in the scalability of scRNAseq [15,16,17], along with major improvements in the spatial mapping and cellular heterogeneity analysis, researchers are making progress in investigating expression levels through entire organs and organisms, to analyze the cellular composition and variability. These large-scale studies have been able to construct cellular maps, for example, of *C. elegans* [18], *D. melanogaster* [13,19] and mouse organs [20,21]. For the human context, Human Cell Atlas [22] is a highly ambitious single-cell mapping project already underway. However, deep challenges in realizing the full potential of scRNAseq have remained. For example, the primary methodology of identification of cells in scRNAseq is by clustering their expression profiles employing a similarity metric [23]. Clustering, in turn, is considerably dependent on how the similarity metric is specified and the feature sets used for defining a profile [24]. A well-known challenge in scRNAseq analysis is the availability of expression values for as few as 10–20 per cent of genes in a given cell [25], while most of the rest are missing values or dropouts due to a small amount of RNA coming from each cell. The background noise also complicates the analyses. These problems pose a great challenge in deciphering the heterogeneity and identity of cells in these populations, posing major analytical barriers in the analysis of scRNAseq data in their full application potential.

Many of the above challenges in scRNAseq data analysis can be effectively addressed by improved computational strategies to cluster single-cell expression profiles in the absence of reliable values for all genes in most of the entities to be clustered. The development of such computational strategies requires rigorous benchmarking on datasets and systems with well-characterized biological contexts. For this purpose, evaluating computational methods for their ability to reproduce topological associations between cells can come handy as one can safely assume that biologically similar cell populations are enriched in terms of their topological similarity to each other. Researchers have hoped that the transcriptome data collected from similar topological locations could be easily labeled by using standard clustering techniques, but it is now clear that such analyses constitute a highly non-trivial problem. As described above, gene dropouts and missing values make these datasets extremely sparse. The missing values belong to different gene sets in each cell and for each measurement of expression profile, further complicating the problem of reconstructing them. To alleviate this problem, and other undesirable attributes of the high-dimensional feature space of scRNAseq data, a priori feature-selection methods are implemented before clustering and downstream analysis of the dataset to identify informative genes to improve clustering results.

The problem of identifying marker genes, which would ultimately contain the complete information of all genes combined and which would be sufficient for further downstream analysis and interpretations have remained challenging and yet of great interest for a long time. For example, one very recently published a study in 2017 by Subramanian et al. [26], has identified approximately 1000 genes which are shown to be sufficient to predict the expression values of remaining genes. Gene-selection methods, like highly variable genes, highly expressed genes and deviance, help identify these informative gene sets which help in better clustering of scRNAseq data. Even many of the computational strategies for spatial mapping of single-cells from scRNAseq data make use of a reference in situ gene expression atlas of several marker genes to rediscover the lost positional information [10,11,12,13,14,27].

Gene (feature) selection is a combinatorial optimization problem, and an exhaustive search of feature space will have to evaluate approximately 2*^N^* different combinations, where *N* is the number of features. Such a course of action will require substantial computational power, or an algorithm which can traverse the vast solution space intelligibly. Genetic algorithm (GA) [28] is one of the most advanced methods for solving combinatorial problems in an efficient and effective manner. It is a metaheuristic that is based on the mechanics of natural genetics and Darwin’s theory of evolution. GA works on a population of individuals to produce successively better “offspring”, by making slight and slow changes (crossover), and often slight changes to its solutions as well (mutation). One of the significant advantages of GA is its ability to search a large solution space and avoid getting trapped in local minima, along with a good convergence rate.

GA has been known to have applications in many fields of science, for example, for solving NP-hard problems, in machine learning, and also in evolving simple programs. A few other of its applications are in neural network designing, like recurrent neural networks, classifier systems and classification algorithms; Traveling Salesman Problem (TSP) and sequence scheduling; robotics; designs; and in economics, as well, like cobweb model and equilibrium resolution. We also see vast applications of GA in medicine, in the areas of proteomics, radiology, infectious diseases, cardiology, healthcare management, haplotype assembly [29], magnetic resonance images [30] and biochemical-parameter estimation and optimization [31]. In general GA is among the battery a set of evolutionary techniques used in feature selection and parameter optimization. In essence GA creates combinations of constituent features (genes) in a population through a selection strategy, thereby doing away with the superfluous and ineffective combinations. This significantly reduces the solution space and the model “evolves” or “learns” to select the most desired combination defined by a fitness function. In this way GA can be used to solve the feature selection, as well as the optimization, problems. Use of GA, for feature selection in gene-expression analysis, has been notably reported in the past. For example, Li L. et al. [32], in 2001, used a Genetic Algorithm/K-nearest neighbor (GA/KNN) coupled strategy to identify genes which can distinguish between several classes of samples from gene-expression data. In 2003, Ooi C.H. et al. [33] tried to determine gene sets and also their optimal sizes, using GA, which maximized classification success. Dolled-Filhart, M. et al. [34] in 2006 identified a set of tissue biomarkers for breast cancer from mRNA profiles using GA. In 2013, Lin T.C. et al. [35] tried to distinguish six subtypes of pediatric acute lymphoblastic leukemia from microarray data by using GA for feature selection, followed by application of silhouette statistics to classify them. Lastly, T. Latkowski et al., 2014 [36] applied GA to select genes which could help in the recognition of autism with high accuracy.

Recognizing the scope of genetic algorithm in improving clustering and classification along with spatial mapping from single-cell transcriptomics data, we participated in Dialogue for Reverse Engineering Assessments and Methods (DREAM) Single-Cell Transcriptomics Challenge [37] to assess whether GA will be a useful technique. DREAM [38] has driven open scientific contests in areas of biology and medicine since their beginning in 2006. DREAM Single-Cell Transcriptomics Challenge (SCTC), is one such challenge where participating teams were asked to predict the positions of cells in the *Drosophila melanogaster* embryo using single-cell sequencing data of 1297 cells from [13], and a reduced number of marker genes from BDTNP database [39], i.e., 60 genes (sub-challenge one), 40 genes (sub-challenge two) and 20 genes (sub-challenge three). The challenge attempts to use fewer marker genes to infer the spatial locations of cells. These challenges are associated with a computational mapping strategy called DistMap [13], developed by Karaiskos et al. [13], in 2017, to reconstruct the *Drosophila melanogaster* embryo. The embryo studied is at the developmental Stage Six, having bilateral symmetry and consisting of approximately 6000 cells which express unique gene combinations. DistMap uses an in situ hybridization pattern of 84 genes from the Berkeley *Drosophila* Transcription Network Project (BDTNP) database [39] with their corresponding 3000 locations, i.e., one-half of the bilaterally symmetric embryo. The algorithm tries to maximize the correlation between single-cell RNA sequencing data and the in situ hybridization pattern of 84 genes in BDTNP. It has been shown that the combinatorial expressions of these 84 genes are enough to map every cell to its position. In order to identify the most important genes out of these 84 and to help model topological locations based on a gold-standard mapping, in this manuscript, we present the use of a genetic algorithm, followed by gene-ontology analysis of selected features. It may be noted that realizing the limitations of benchmarking presented in this challenge, the term “silver standard” was finally employed in the published consortium paper. We have in this paper used gold and silver standard terms equivalently but essentially refer to the DREAM challenge benchmarks, on which different methods have tried to perform the best.

## 2. Method

### 2.1. DREAM Dataset Description

The reference database used to develop and benchmark GA-based feature selection for gene-expression profile clustering is taken from DREAM single-cell challenge, as explained below. The dataset consists of expression pattern of 84 marker genes obtained from in situ hybridization experiments. The database comes from BDTNP [39] and available as *bdtnp.txt*. The expression pattern corresponds to 3039 locations, i.e., one-half of the bilateral symmetric *D. melanogaster* embryo at Stage 6. The 3039 locations are defined by x, y and z coordinates, which are in the same order in the reference database and available as *geometry.txt*. The single-cell gene-expression data are obtained from embryos at Stage 6 of development. Both raw and normalized UMI counts are available of 8924 genes from 1297 cells and are available as *dge_raw.txt* and *dge_normalized.txt* respectively.

For predicting the location of 1297 cells using 84 marker genes we use DistMap. Firstly, instead of using the continuous gene-expression data from BDTNP, the binarized data is used which was done by selecting a threshold for each gene individually [13] and is available as binarized_bdtnp.txt. Similarly, scRNAseq data are binarized by using binarizeSingleCellData() function in DistMap [13], which binarizes by using a quantile threshold of 0.23, obtained through minimization criteria. The mapCells() function calculates a confusion matrix for each cell–bin combination. Mathews correlation coefficient (MCC) was used to weight confusion matrices and each cell–bin combination was assigned a score. The MCC matrix obtained by using all 84 marker genes to map cells is considered as the gold standard MCC.

Datasets and additional challenge details are available for download at https://www.synapse.org/#!Synapse:syn16782360 after registration at Synapse for free.

### 2.2. Selection of Gene Sets

Selecting *n* features (genes) out of 84 known biomarker features is a combinatorial problem of high complexity in which ^84^C_n_ combinations are possible. Genetic algorithm (GA) is a well-known technique for solving combinatorial problems at a low computational cost. The critical requirement for applying GA is the choice of a fitness function. In biological systems, we know that optimization of one scoring metric may leave other measures of performance inadequately optimized. Hence, we first identified a set of four metrics which represent various aspects of model performances and designed a framework in which GA could simultaneously achieve optimization of all of them. After defining the GA training framework, identical protocols were applied to select different sizes of target subsets, i.e., of size 60, 40 and 20, corresponding to DREAM sub-challenges 1 to 3.

### 2.3. Data Preprocessing

In a preliminary experiment, we tested if some commonly used imputation methods will improve the cell topology predictions and our results were negative (Detailed provided in Supplementary Materials Table S1). In brief, we used the original DistMap algorithm to predict topological associations of all the given cells with expression profiles of 84 features as explained above. We computed the MCC of the binarized locations of topological information with and without imputing for the missing expression values, employing some of the most popular methods for imputing scRNAseq missing values. The supplementary data in Supplementary Materials Table S1 shows that the imputation of missing values by either of these methods did not significantly alter the MCC values obtained from the raw values in which missing values are simply replaced by zero before computing these scores. Based on this preliminary assessment, we used all the raw datasets without any preprocessing for further feature selection and DREAM challenge questions.

### 2.4. Training Model

Selection of the best subset included in our submission to DREAM sub-challenges was achieved in two steps, viz (1) GA training and (2) gene ontology (GO) based prioritization. GA is used to produce data-driven candidate sets which learn to reproduce the outcomes of the cell to bin correlations from a subset of genes. Step 2 ensures that the final sets are biologically consistent with the original driver gene sets. Details are explained in the following sections.

#### 2.4.1. Genetic Algorithm

Genetic-algorithm-based optimization is performed using a customized code developed for this very purpose from scratch and implemented in the R programming language. In-house customization of code was helpful in implementing restrictions on population size and other parameters more conveniently as detailed further.

#### 2.4.2. Fitness Function

The Mathews correlation coefficient (MCC) matrix calculated by DistMap using all 84 genes, which contains MCC scores between binarized in situ hybridization expression values and location-based cellular assignments for each cell–bin combination is assumed as the gold standard MCC matrix (*M*). We have defined a fitness function which minimizes the difference with the gold standard using four metrics, each of which aims to quantify this difference or similarity differently.

#### 2.4.3. Metric-1 Based on Root Mean Squared Deviation

In this particular metric, using the MCC matrix obtained using 84 driver genes from DistMap as the gold standard for the problem, our GA-model tries to minimize the error, i.e., the difference between MCC obtained by selecting a subset of genes (matrix *N*) and MCC, obtained by using 84 genes (matrix *M*).

(1) $$s_{1}=\frac{\sum_{i=1}^{a}{\left(M-N\right)}^{2}}{a},$$

where *M* corresponds to MCC matrix calculated with *n* = 84. *N* corresponds to MCC values for subset *n* = 20 or 40 or 60; *a* = 1297 × 3039 corresponding to each element in MCC matrices.

#### 2.4.4. Metric-2 Based on Spearman Correlation

Entire matrix differences as a fitness function of GA is not complete because the far off distances are weighted equally as nearby pairs of topological positions, which is not biologically desirable. This is achieved by taking the Spearman correlation between MCC values of each cell produced by 84 driver genes and those provided by a candidate subset. Contrary to Metric-1, Spearman correlation should increase with fitness, so we modified the Spearman’s correlation in the negative direction as follows:

(2) $$s_{2}\;=[\;2-\;\sum_{i=1}^{1297}\frac{\sum_{b=1}^{3039}\left(\;R\left(M_{i}^{b}\right)-\underline{R(M_{i})}\right)\;\left(\;R\left(N_{i}^{b}\right)-\underline{R(N_{i}}\right))}{\sqrt{\;\sum_{b=1}^{3039}(\;R\left(M_{i}^{b}\right)\;-\underline{R(M_{i}}){)}^{2}\;\sum_{b=1}^{3039}{\left(\;R\left(N_{i}^{b}\right)-\underline{R(N_{i})}\right)}^{2}}}]$$

where *R*(*x*) corresponds to the vector of ranked values in *x*; *M* corresponds to MCC matrix, calculated with *n* = 84; *N* corresponds to MCC values for subset *n* = 20 or 40 or 60; and *a* = 1297 × 3039, corresponding to each element in MCC matrices.

As a result, the new Metric-2 also needs to be minimized during GA learning.

#### 2.4.5. Metric-3 Based on Jaccard Index

Spearman correlation as defined in Metric-2 tries to rank all predicted subsets to improve MCC–MCC relationships. However, as the correct position of a cell should be in at least top 10 candidate cell positions predicted, we also the third scoring function viz a Jaccard index as the intersection of highest scoring bins (from 84 driver genes) to predicted top 10 bins (from candidate subsets). The sign reversal was performed similarly to Metric-2 to ensure that fitness function is minimized consistently with Metric-1 and Metric-2. Thus, this scoring metric is defined as follows:

(3) $$s_{3}\;=\frac{\sum_{i\;=\;1}^{1297}\left(2\;-\frac{n\left(\;P_{i}\cap Q_{i}\;\right)}{n\left(P_{i}\right)}\right)\;}{1297},$$

where *P* corresponds to a matrix of assigned bins for each cell according to highest MCC scores in matrix *M* (*n* = 84); *Q* corresponds to a matrix of top 10 assigned bins for each cell according to highest MCC scores in *N* (*n* = 20/40/60).

#### 2.4.6. Metric-4 Based on Euclidean Distance

The three scoring functions defined above, represent MCC–MCC relationships between similarities predicted by 84-probe set versus a selected feature set. However, exact topological position of each cell is known and GA model may try to optimize the model’s ability to correctly assign each cell to its location of origin. To consider topological properties of the cell, we calculated Euclidean distances between the best-predicted bin from all 84 genes to the best-predicted bin from the selected subset of genes.

(4) $$s_{4}=\sqrt{{\left(x_{P_{i}^{1}}-x_{Q_{i}^{1}}\right)}^{2}{\left(y_{P_{i}^{1}}-y_{Q_{i}^{1}}\right)}^{2}{\left(z_{P_{i}^{1}}-z_{Q_{i}^{1}}\right)}^{2}},$$

where $P_{i}^{1}$ corresponds to *i*th cells top most predicted bin in matrix *P* and similarly for *Q*.

By default, this function also needs to be minimized as previous scoring metrics. This score differs from Metric-1 in the sense that only the best-predicted bin is compared here with the expected bin position.

#### 2.4.7. Final Fitness Function

As all the four metrics are modified to be minimized and non-negative and non-zero, the fitness function was defined as the geometric mean of all four metrics defined above. We assigned single cells to their respective location using reduced gene sets of size 20, 40 and 60. The genetic algorithm selected the best gene set for each target set size.

#### 2.4.8. Parameters

Unlike the standard genetic algorithm packages (such as *genealg* and *ga* in R) for which implementations were readily available, we did not try to perform binary feature selection. Instead of taking a binarized set of features, in which the number of features is also determined by GA, we hard-coded the selected number of features at *n* = 20 or 40 or 60 as required in each sub-challenge Hyper-parameters of GA were selected by doing a few hit and trial experiments to ensure smooth learning curves for GA iterations. The four parameters were determined as follows:

- Initial population:
  Initial population was a set of sequences of length *n*, carrying indexes of different columns we need to incorporate. Hence, we could set the exact length of the number of features to be selected by this. The initial population is defined by randomly generating 500 chromosomes of feature indexes of size *n*.
- Crossover:
  A crossover is done between two chromosomes in the population, generating two offspring. A single point crossover is done between two randomly selected chromosomes, at a randomly selected point one minus the length of the chromosome. The chromosomes are further crossed over if the offspring chromosomes do not have a repetition of gene sets and added to the next generation. 200 offspring are generated by crossing over chromosomes 100 times.
- Mutation:
  A mutation is introduced in the population set every third generation. For every chromosome in the generation, a location is randomly selected and replaced with a new feature index not already present in the chromosome.
- Elitism:
  At the end of each generation, we selected only the top 20% of the offspring as parents for the next generation.
- Gene Ontology Analysis:
  The GA-based approach is a simple data-driven approach which may not produce the most biologically meaningful results. For this purpose, GA optimized top sets (from last few generations of GA) were re-prioritized to ensure that the pathway enrichment in the final set of genes also has the best overlap with the enrichments produced by 84 driver genes. Hence, the last three unique gene sets obtained for each subset size were analyzed for gene set/GO-based enrichment analysis, using the FlyMine enrichment tool [40]. The set with the maximum overlap of GO terms was selected as the final set of genes.

### 2.5. Post Competition Assessment of GA Hyperparameters

After the DREAM competition results were announced we did a mild assessment of fine tuning GA that could have potentially impacted the outcomes of GA-derived features. Several experiments were conducted for this purpose. In the first, we used different seeds for GA and evaluated the variance/robustness of predicted scores. Secondly, we systematically varied some of the GA hyperparameters and assessed how the crossover rate, mutation rate and elitism could potentially impact the performance of GA for location prediction.

### 2.6. Creating Baseline Gene Sets to Evaluate Performance Gains in a Complex Method

To compare our results, we generated three kinds of additional gene sets, using other simpler approaches. First, we generated a protein–protein interaction network on STRING v11 [41] by using all known interaction sources in the database. Network analysis was done using Cytoscape 3.8.0 [42] and genes with the highest degree were selected to create gene sets with top 20, 40 and 60 genes. Secondly, gene variance analysis was done using Seurat v3.2.1 [14] FindVariableGenes function with a variance-stabilizing transformation method [43,44]. Using standardized variances generated, we selected the top most variable and least variable (*stable*) gene sets of size 20, 40 and 60. Thus variable (VAR), stable (STB) and protein–protein interaction network (PPI)-degree (PPI) are the three criteria used to shortlist genes for comparison with GA-based methods producing similarly sized feature sets.

### 2.7. Evaluating Performance of Selected Gene Sets by Comparing with Different Location Prediction Methods

As mentioned, DREAM challenge can be thought of being made of two components. First is that of feature selection in which 20, 40 or 60 features out of 84 well-known topological predictors are selected. These selections may either try to reproduce the topologies predicted by 84 features or may go all the way to do a direct topological prediction, perhaps even better than the 84 features. Since the final evaluation is carried out in an integrated manner, it is not always obvious if a method is performing well due to feature selection or topological prediction. As shown in Supplementary Table S2, a variety of methods focusing on either of this goal were employed by competing teams. In our case, we tried to combine the features selected by our GA with the topological prediction method of the best performing team (post competition) to assess if the results could have been even better if a hybrid approach like this were to be used. Specifically, this location assignment, proposed by Team Thin Nguyen in this approach is performed by predicting only one instead of all 10 desired locations and then assigning the nearest 9 bins based on the best selected single location.

## 3. Result

### 3.1. GA Optimization of Fixed Sized Gene Sets

**Figure 1:**
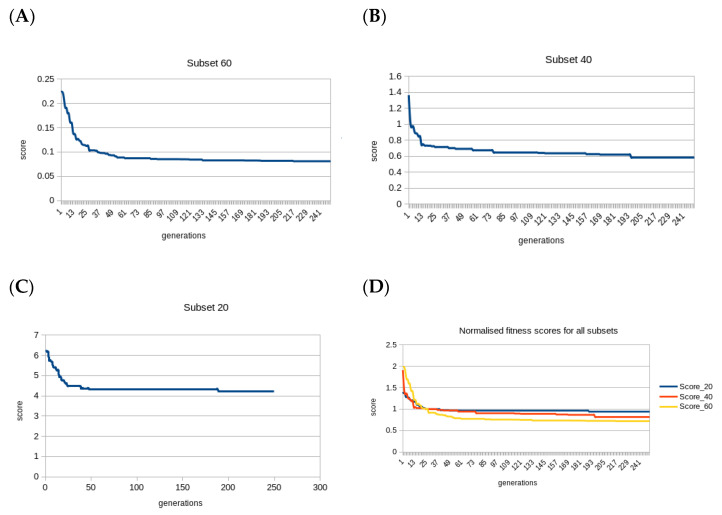
Learning curve (best score in each generation) of genetic algorithm (GA) feature selection with (**A**) 60 features (**B**) 40 features and (**C**) 20 features as the gene-set size to be optimized. Fitness function stabilizes gradually for all three subsets within 250 generations approximately. The fitness function scores were median normalized for the three subset sizes and show a very similar curve (**D**).

To avoid over-fitting and also keep it computationally less expensive, different hyperparameters of GA were only varied slightly. Thus, the mutation rates were tried between two and four, of which three gave better results than the mutation rate of two or four. The number of offspring was selected to be 200, as good convergence was observed with this without need to tweak it further. Elitism was kept at 20%, based on our experience of dealing with similar problems (no parameterization was attempted for the number of offspring and elitism). During training, even though the fitness function was a hybrid of four scores defined above, all four metrics were found to be optimized individually with the best score, giving an almost smooth learning curve (Figure 1).

The fitness function was systematically optimized and reached its minima in a relatively smooth curve, after approximately 250 generations for all three simulations. All four component metrics in the fitness function were successfully optimized. The last three unique gene sets after the GO-based enrichment analysis showed that the overlap of GO terms improved consistently with the increase in the number of iterations, except for *n* = 60, wherein the third-best set showed the best overlap with driver genes in terms of GO terms.

The MCC matrix obtained from selected genes was sorted, to obtain top 10 locations for each cell and was submitted in each sub-challenge. The scoring was done based on three scores which were not disclosed until the end of the competition. The scores measured the accuracy of location predicted, correlation of predicted location with expression from reference atlas, variance of predicted location and the extent to which original spatial patterns were reconstructed [37]. The gene set selected using GA are listed in Table 1 and their corresponding scores in Table 2.

### 3.2. Ranking of Proposed GA Method in DREAM Challenge and Post-Competition Experimentation

As per the scores announced by the DREAM organizers, our team, codenamed “SciWhyGeeks”, scored on the 8th, 7th and 12th position, among the 38 participating teams in the final stage. These scores are impressive, given that the approach was purely data-driven and scalable to many different types of similar problems. Post-competition, we performed a number of experiments to gain insights into the GA implementation for feature selection. First of all, we compared the runtime of GA in comparison to some of the top methods for which source codes were available and could be implemented without a need for additional/unavailable libraries. Supplementary Table S3 summarises results from 5 methods in addition to GA and suggests that GA, being supervised method takes longer than some unsupervised techniques but is competitive to other supervised methods. The advantages of a supervised method despite being slower are discussed in the later sections (see discussions). For the performance level assessments, we retrained GA models with three additional initial seeds each to evaluate the stability of the finally selected features under local minimization. This ensures the generalizability of final scores on new applications of the method. Secondly, we evaluated if a double crossover GA implementation could have improved the topological assignments by a better search of the solution space. Finally, we analyzed the performance of our implementation of GA in terms of the integrated versus individual fitness functions, used in our original submission. We repeated our feature-selection methods on each fitness function individually and then compared it with the complex hybrid fitness function that we originally employed.

**Figure 2:**
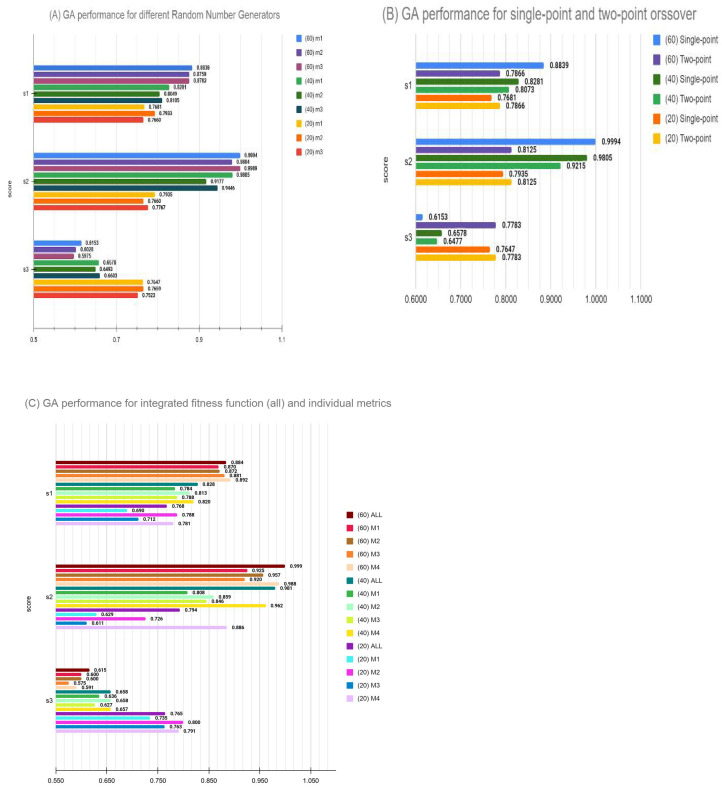
Barplot showing comparison of three Dialogue for Reverse Engineering Assessments and Methods (DREAM) sub-challenge score (s1, s2 and s3) of gene sets generated for (**A**) different random number seeds (rn1, rn2 and rn3). (**B**) Single-point and two-point crossovers. (**C**) Integrated fitness function and each metric individually. Scores generated after bootstrapping 1000 times, and definitions are as provided by DREAM Single Cell Transcriptomics Challenge. These three scores are related to the accuracy of location predicted, correlation of predicted location with expression from reference atlas, variance of predicted location and the extent to which original spatial patterns were reconstructed [37].

Figure 2 shows the results from these three experiments. In Figure 2A, it is observed that the three different seeds to initiate GA training do make a small difference in each of the three benchmarking scores of the SCTC challenge. Even though these differences are only a small percentage of the overall scores, in a tight competition such as this, final rankings may get randomly influenced a bit by this noise. We therefore believe that the top ranking of feature selection cannot be a rigid gold standard for feature-selection methods, and some margin of error has to be accounted for due to the uncertainty in converged solutions. In Figure 2B, we observe that two-point crossover results were consistently outperformed by single-point mutations, as used in the original submission. Thus, a minimalist GA-based feature selection was found to be superior at least in terms of crossover-selection strategy. Finally, we evaluated our integrated fitness function, which was composed of the geometric mean of four individual fitness functions. We observed that, even though the objective of GA optimization was to maximize integrated fitness, the component fitness function saw a consistent optimization (see Figure 2C). Thus, for example, the performance of integrated fitness function was best four times and second-best three times out of a total of nine comparisons. Next we see M4 performing best two times and second-best five times out of a total of nine comparisons. M2 is also seen to perform best three times out of nine comparisons. M1 performed best only once, and M3 did not perform best in any of the nine comparisons. In summary, we conclude that the scoring system employed here can be used a reliable approach to employ complex objective functions in GA-based feature selections.

### 3.3. Feature Selection versus Location Assignment

**Figure 3:**
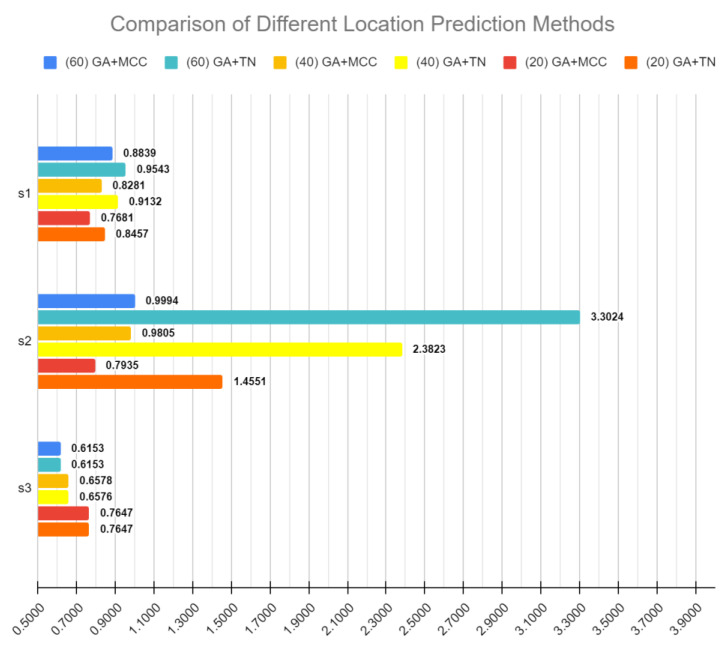
Barplot showing comparison of three DREAM sub-challenge score (s1, s2 and s3), using a different location prediction method, on gene sets generated by GA feature-selection algorithm. GA + Mathews correlation coefficient (MCC) and GA + Thin Nguyen (TN). TN is the location prediction method used by top-performing team in Sub-Challenge1 (Thin Nguyen). Scores generated after bootstrapping 1000 times and definitions are as provided by DREAM Single Cell Transcriptomics Challenge.

**Figure 4:**
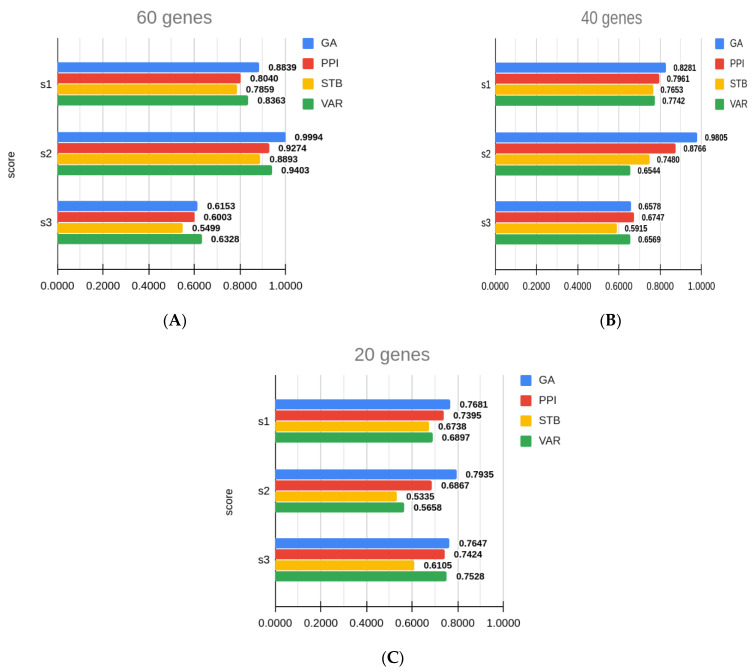
Barplot showing comparison of three DREAM sub-challenge score (s1, s2 and s3 are shown in (**A**), (**B**) and (**C**) respectively) of gene sets generated by different methods (GA = genetic algorithm, PPI = protein–protein interaction network, STB = least variable genes (stable), VAR = most variable genes) after bootstrapping 1000 times. Score definitions are as provided by DREAM sub-challenge.

As discussed above, SCTC effectively can be thought of a two-step process viz (a) feature selection of 84 that can best represent all of their predictive capabilities, and (b) the topological assignment strategies after the features have been selected or even over the 84 features overall. Our GA-based approach used an integrated fitness function and employed DistMap strategy for topological assignment. The top scoring team, however, adopted a strategy of only predicting the most plausible topology and assigning the remaining nine positions of the bins by simply finding their nearest locations. We investigated (post-competition) if employing the topological assignments of the best performing team over our selected features could have outperformed the predictions and perhaps did even better than the best-performing strategy. Figure 3 shows the results of these experiments in detail. We do observe that the best-performing approach of SCTC, called Thin Nguyen (TN) in this annotation, was indeed crucial and when combined with GA-based features selected by our method outperforms all the scores, but most significantly the score 2 of DREAM challenge in the case of 60 feature selections. More details of each of combining TN-based locations with other competitors are shown in Table 3, graphically presented in Figure 4. From the analysis of these scores, we conclude that GA remains the best-performing feature-selection strategy, whereas the best-performing team’s approach to utilize only the best location prediction and then extrapolating them to nearby locations was a key winner for them in getting the best position.

### 3.4. Comparison with Other Gene Sets

As described in the Methods, to understand the utility of GA as compared to trivial feature selection approaches, we defined three additional simple criterion of subset selection and re-scored their performance for the benchmarks provided by DREAM sub-challenges. The three approaches for selecting these gene sets are as follows: (1) gene set based on highest variability across single cells out of 84 genes already known to be biomarkers. (VAR). (2) gene set based on least variability or being the most robust or stable genesets (STB). (3) Most connected protein–protein interaction networks modules from 84 genes, by selecting top *n* genes from 84 genes based on their highest connectivity scores. Connectivity scores are computed by first creating a PPI for the 84 genes and from the PPI networks, selecting the nodes with highest degree (number of connections to any other gene with the set of 84). Results of these experiments are presented in Table 4. 

We scored the other gene sets using the same three-score criteria used in the challenge. We see that GA-based gene set selection performs noticeably better for s2 score which is based on correlation of predicted location, comparably with other gene sets for s1 and s3 score which is based on accuracy of location predicted and spatial pattern reconstruction, respectively.

### 3.5. Parameter Evaluation Post DREAM Challenge

As stated in Methods, we carried out experiments to assess the performance of GA under the following conditions:

(a) The initialization conditions and GA robustness: In these experiments, we performed 10 runs of GA optimization with random initialization every time. Overall, 30 experiments (10 for each of the 20, 40 and 60 feature sets) were carried out, and three scores, namely s1, s2 and s3, were compared. Overall, 90 comparisons shown in Supplementary Table S4 and represented in Supplementary Figure S1 clearly indicate the GA has successfully avoided local minima as the variance in 10 runs in each case is less than 5% of the mean performance score with the average variance across all runs being less than 2%. This demonstrates that GA algorithm can be safely employed for a few runs to get an optimal solution when the computing cost for feature selection is too high.
(b) Variation of performance was also examined by a combination of crossover and mutation rates, as well as elitism in the model. Our original model used 100% crossover with no parents allowed to cross over for a faster convergence within the time limit of DREAM challenge. Post-challenge, we examined these variations and found that, for the small feature set target of 20 genes, results are not too sensitive to hyperparameters. However, larger feature set selections are somewhat unstable and a combination better than our challenge submission did exist. Thus, we conclude that GA optimization may benefit from larger scan of hyperparameter space when permissible. Nonetheless, an intuitively selected set of hyperparameters did perform well enough to remain competitive in the blind competition.

## 4. Discussion

Gene sets with reduced sizes were able to recover the original cell–bin relationship efficiently, as indicated by strong MCC–MCC and topology-assignment scores. The reduced gene set is able to capture the overall intrinsic relationships between these functionally related genes. Applying genetic algorithms to identify the reduced gene sets helps us span through the whole sample space of possible solutions, of high complexity, in an efficient manner. It is known that the gene expression interactions are complex and deeply interactive. Such intrinsic correlation in gene expression can be exploited to mine unique patterns which can help solve and improve similar challenges that still exist. There are a few limitations in this method. The base algorithm of DistMap is used to generate the optimal cell–bin relationship with a complete gene set and gene subsets, but it has its weaknesses. One of them is that it uses binarized data, which cause a loss of information contained in both the single-cell gene expression data and BDTNP. The classical genetic algorithm is used to select the optimum number of genes. However, we hard-coded the optimum size in this case. Hence the user can define the number of features they wish to have. Further improvement in the selection, made by using the genetic algorithm, can be made by scanning a wider sample space, using a broad range of parameters, along with the implementation of cross-validation techniques.

One question that may arise in the use of current GA-based approach is that it is a supervised technique, like a few other top-rated teams in DREAM challenge. For example, Random Forest and Particle Swarm Optimization have been used effectively for breaking the teams into top 10 in some of the sub-challenges. On the other hand, purely unsupervised feature selections based on expression variability (and also principal components analysis) have been shown to be particularly successful in this DREAM challenge [45]. In Supplementary Table S3, we shows that, as expected, the time taken by an unsupervised feature selection is much less than GA or particle swarm optimization. However, while unsupervised methods can produce the most informative features out of a set, in a general context, they cannot operate together with any specific objectives. Supervised methods incorporate an objective function (fitness function in GA). We have clearly observed that optimization of one scoring function leaves others in a sub-optimal state. For example, optimizing models for location prediction may not produce the topologies desired. Similarly, if one would like to know the best feature set that can describe a given phenotype, such as one disease or the other, unsupervised techniques will not be able to produce distinct sets without introducing additional steps. Therefore, development of both supervised and unsupervised techniques for feature-selection and goal-seeking models are needed, of which GA, as one example, was discussed in this work.

## 5. Conclusions

Successful implementation, DREAM official scores and additional analysis point out that the genetic algorithm helped identify a smaller subset of genes which could uncover the original intrinsic pattern of the cell–bin relationship. There are vast applications of the genetic algorithm across various fields, and here too, by remodeling the algorithm to this specific problem, wherein looking for an optimal solution can be like looking for a needle in a haystack.

## Figures and Tables

**Table 1:** Final subset of genes identified for each *n*.

*n* = 60	*aay, Ama, Ance, Blimp-1, bmm, brk, Btk29A, bun, cad, CG10479, CG11208, CG14427, CG43394, CG8147, croc, Cyp310a1, D, dan, danr, Dfd, Doc2, edl, erm, eve, fj, fkh, ftz, gk, gt, h, hb, Ilp4, ImpE2, ImpL2, kni, Kr, lok, Mes2, MESR3, mfas, NetA, noc, nub, numb, oc, odd, prd, pxb, rau, rho, run, sna, tkv, tll, toc, Traf4, trn, tsh, twi, zen*
*n* = 40	*Ama, Antp, Blimp-1, brk, Btk29A, CG10479, CG43394, CG8147, danr, disco, Doc3, edl, eve, fkh, ftz, gt, h, Ilp4, ImpE2, ImpL2, ken, kni, lok, Mdr49, MESR3, Nek2, noc, nub, numb, oc, pxb, rau, rho, run, sna, toc, Traf4, trn, tsh, twi*
*n* = 20	*brk, CG43394, CG8147, dan, Doc2, h, Ilp4, ImpL2, kni, Kr, Mdr49, MESR3, Nek2, noc, oc, odd, rho, sna, trn, tsh*

**Table 2:** Scores and rank for the three challenges by our team in the challenge, using genesets in Table 1.

Sub_Challenge	Subset_Size	Score 1	Score 2	Score 3	Rank
1	60	0.6991 ± 0.0057	0.9997 ± 0.0057	0.6142 ± 0.0057	8
2	40	0.6532 ± 0.0064	0.9815 ± 0.0064	0.6572 ± 0.0064	7
3	20	0.6137 ± 0.0083	0.7943 ± 0.0083	0.7646 ± 0.0083	12

**Table 3:** Scores of GA + TN method in comparison with the top 10 teams in the challenge.

Subset	Name of the Team	S1	S2	S3	Rank
**60**	**GA + TN**	(0.7929)	(3.3008)	0.6141382	-
	Thin Nguyen	((0.776))	((3.0637))	0.6178	1
	WhatATeam	0.7002	1.5327	(((0.6268)))	2
	NAD	0.7504	1.6614	0.5916	3
	Christoph Hafemeister	0.667	1.0624	(0.6506)	4
	zho_team	(((0.7663)))	(((2.5973)))	0.5635	5
	MLB	0.6826	1.0245	0.6469	5
	OmicsEngineering	0.6738	1.0188	0.6258	6
	Challengers18	0.661	1.4522	0.6122	10
	DeepCMC	0.6668	1.0194	0.6271	10
	BCBU	0.6506	1.2276	0.6037	13
**40**	**GA + TN**	(0.7442)	(2.3817)	0.6570	-
	WhatATeam	(((0.6869)))	1.164	0.6672	1
	OmicsEngineering	0.6511	0.9991	((0.6899))	2
	NAD	((0.7367))	(((1.4341)))	0.5968	3
	Christoph Hafemeister	0.6587	0.976	0.6837	4
	Challengers18	0.6552	1.3176	0.6538	4
	MLB	0.647	0.909	(0.7076)	5
	DeepCMC	0.6524	0.9846	(((0.6846)))	6
	zho_team	0.6657	((1.6043))	0.5353	9
	BCBU	0.625	1.1918	0.6241	11
	Thin Nguyen	0.6265	1.258	0.5836	12
**20**	**GA + TN**	((0.6923))	(1.4546)	0.7646	-
	OmicsEngineering	0.6554	0.9534	(((0.7934)))	1
	NAD	(0.7217)	((1.2445))	0.6534	2
	Challengers18	0.662	1.0166	0.7928	2
	WhatATeam	0.6504	0.9327	0.7783	3
	DeepCMC	(((0.6621)))	0.8411	(0.818)	4
	BCBU	0.6406	(((1.1456)))	0.6393	5
	MLB	0.642	0.7579	((0.8156))	7
	Thin Nguyen	0.6462	0.8791	0.6302	9
	Christoph Hafemeister	0.6017	0.9056	0.6341	14
	Zho team	0.5234	0.8545	0.4546	18

Cells highlighted in single, double and triple brackets represent the top three ranks within each challenge, respectively. Source for Thin Nguyen (TN) team’s prediction method (Sub-challenge 1) is available from https://github.com/thinng/exp2loc.

**Table 4:** Scores of different gene sets after bootstrapping 1000 times.

Sub_Challenge	Subset_Size	Method	Score 1	Score 2	Score 3
1	60	GA	0.8839 ± 0.0056	0.9994 ± 0.0191	0.6153 ± 0.0042
	60	PPI	0.8040 ± 0.0075	0.9274 ± 0.0275	0.6003 ± 0.0050
	60	STB	0.7859 ± 0.0076	0.8893 ± 0.0188	0.5499 ± 0.0043
	60	VAR	0.8363 ± 0.0074	0.9403 ± 0.0319	0.6328 ± 0.0047
2	40	GA	0.8281 ± 0.0065	0.9805 ± 0.0247	0.6578 ± 0.0037
	40	PPI	0.7961 ± 0.0068	0.8766 ± 0.0259	0.6747 ± 0.0058
	40	STB	0.7653 ± 0.0088	0.7480 ± 0.0215	0.5915 ± 0.0042
	40	VAR	0.7742 ± 0.0082	0.6544 ± 0.0206	0.6569 ± 0.0066
3	20	GA	0.7681 ± 0.0088	0.7935 ± 0.0245	0.7647 ± 0.0040
	20	PPI	0.7395 ± 0.0122	0.6867 ± 0.0299	0.7424 ± 0.0065
	20	STB	0.6738 ± 0.0133	0.5335 ± 0.0211	0.6105 ± 0.0060
	20	VAR	0.6897 ± 0.0114	0.5658 ± 0.0305	0.7528 ± 0.0079

## Data Availability

Data sets used in this study are available from https://www.synapse.org/. Source codes for the GA-based programs used in this work will be shared via email and will be uploaded to the group website www.sciwhylab.org when documentation to its usage is ready.

## Supplementary Materials

The following are available online at https://www.mdpi.com/2073-4425/12/1/28/s1, Table S1: Correlation of MCC scores before and after imputation. Topological associations were predicted from 84 features in BDTNP data set with and without imputation of missing values in all scRNAseq values. MCC values from different methods produced worse or similar scores when well known imputation techniques were used to reconstruct missing values. Table S2: Summary of most successful methods in DREAM SCTC challenges. This table summarizes overall list of techniques used by various competing teams. Exact team-wise details and individual methods used can be accessed from DREAM website and the consortium paper. The methods are grouped into feature selection methods and location prediction methods, as discussed in the manuscript. Table S3: Runtime of GA in comparison with 5 of the top 10 teams. Among the supervised methods time taken by GA is slightly less than taken by Particle Swarm Optimization (Chalennegers18) but significantly slower than Random forest, which ranked relatively lower in 2 of three sub-challenges. It is not clear if the gain in time was indeed due to the techniques or a better implementation as they mostly employed highly optimized publicly distributed packages, whereas our GA was implemented from the scratch. Table S4: Performance of GA under different hyper-parameter settings for reproducing location coordinates of single cells in DREAM challenge. GA-0 refers to the original parameters used for DREAM challenge and GA-x refers to different arbitrarily selected parameters to assess the sensitivity of performance levels towards these parameter settings. Scores s1, s2 and s3 are the different metric used by DREAM organizers to assess performance levels, as described in the manuscript. A total of 12 combinations have been tested and show that the GA-0 has a competitive performance despite keeping a high cross-over rate and an intuitively selected set of parameters for the challenge. Figure S1: Assessment of robustness of GA models under different initialization conditions. A total of 10 runs are attempted for each GA model and performance scores s1, s2 and s3 for each the target feature size is shown as a single point in the plot. Parameter settings for all these runs are as used in the original DREAM submission. Overall variance in most GA runs is less than 5% of the mean value of the score with average variance across all runs being just 2 percentage points, suggesting that the GA initialization does not impact the final model performance and the trained models are highly robust in terms of their predictive performance levels.
