# Supplementary material for: Feature Selection for Topological Proximity Prediction of Single-Cell Transcriptomic Profiles in Drosophila Embryo Using Genetic Algorithm
Source: Genes (Basel). 2020 Dec 28;12(1):28. doi: 10.3390/genes12010028 (PMC7824175; doi:10.3390/genes12010028)
Supplement: Supplementary file 1 [file genes-12-00028-s001.pdf]

Supplementary Table S1: Correlation of MCC scores before and after imputation. Topological associations were predicted from 84 features in BDTNP data set with and without imputation of missing values in all scRNAseq values. MCC values from different methods produced worse or similar scores when well known imputation techniques were used to reconstruct missing values.

	MCC_mean	Correlation with original
**Original DREAM submission**	1.137429	NA
**Scnorm [1]**	1.145078	0.9805988
**Saver [2]**	1.020666	0.7694661
**Scimpute [3]**	1.049111	0.7670297

1. Bacher, R., Chu, L., Leng, N. et al. SCnorm: robust normalization of single-cell RNA-seq data. Nat Methods 14, 584–586 (2017). https://doi.org/10.1038/nmeth.4263

2. Huang, M., Wang, J., Torre, E. et al. SAVER: gene expression recovery for single-cell RNA sequencing. Nat Methods 15, 539–542 (2018). https://doi.org/10.1038/s41592-018-0033-z

3. Li, W.V., Li, J.J. An accurate and robust imputation method scImpute for single-cell RNA-seq data. Nat Commun 9, 997 (2018). https://doi.org/10.1038/s41467-018-03405-7

Supplementary Table S2: Summary of most successful methods in DREAM SCTC challenges. This table summarizes overall list of techniques used by various competing teams. Exact team-wise details and individual methods used can be accessed from DREAM website and the consortium paper. The methods are grouped into feature selection methods and location prediction methods, as discussed in the manuscript.

Selection Methods	Prediction Methods
**SFR (Supervised Feature Ranking)**<br>Random Forest, LASSO, Neural Network<br>Feed-forward Neural Network, Genetic Algorithm,	**CMB (Combination model prediction with MCC)**<br>Random Forest, Feed-forward Neural Network, Local Outlier Factor
**UFR (Unsupervised Feature Ranking)**<br>Particle Swarm optimisation, PCA, Expression Correlation, Stepwise regression, K-nearest neighbours, F-score, Non-negative Discriminative Feature Selection, Hierarchical Clustering	**SIM (Similarity measure - non-MCC)**<br>Weighted correlation, Correlation, F-score, hamming distance, Silhouette score
**KNW (Background knowledge)**<br>Clustering	**MCC (Matthews Correlation Coefficient)**
**VAR (Variance)**	

Supplementary Table S3: Runtime of GA in comparison with 5 of the top 10 teams. Among the supervised methods time taken by GA is slightly less than taken by Particle Swarm Optimization (Chalennegers18) but significantly slower than Random forest, which ranked relatively lower in 2 of three sub-challenges. It is not clear if the gain in time was indeed due to the techniques or a better implementation as they mostly employed highly optimized publicly distributed packages, whereas our GA was implemented from the scratch.

**Team**	**SciWhyGeeks**	**Thin Nguyen**	**Christoph Hafemeister**	**BCBU**	**Challengers18**	**MLB**
**SC1 Rank**	8	1	4	13	10	5
**SC2 Rank**	7	12	4	11	4	5
**SC3 Rank**	12	9	14	5	2	7
**Language**	R	Py	R	R	Matlab	Matlab
**Feature selection method**	Genetic Algorithm	Variance, Non-negative Discriminative Feature selection	Principal component analysis on most variable genes, Expression correlation	Random Forest	Particle Swarm Optimization	Stepwise regression, PCA, k-nearest neighbors, F-score
**Time taken**	1079 min	< 5 min	1 min	10 min	1155 min	149 min

Supplementary Table S4: Performance of GA under different hyper-parameter settings for reproducing location coordinates of single cells in DREAM challenge. GA-0 refers to the original parameters used for DREAM challenge and GA-x refers to different arbitrarily selected parameters to assess the sensitivity of performance levels towards these parameter settings. Scores s1, s2 and s3 are the different metric used by DREAM organizers to assess performance levels, as described in the manuscript. A total of 12 combinations have been tested and show that the GA-0 has a competitive performance despite keeping a high cross-over rate and an intuitively selected set of parameters for the challenge.

		**GA parameter sets**											
		**GA-0**	**GA-1**	**GA-2**	**GA-3**	**GA-4**	**GA-5**	**GA-6**	**GA-7**	**GA-8**	**GA-9**	**GA-10**	**GA-11**
Parameter setting	**Iterations**	250	250	250	250	250	250	250	250	250	250	250	250
	**Initial Population**	500	500	500	500	500	500	500	500	500	500	500	1000
	**Elitism**	0.20	0.20	0.20	0.25	0.15	0.20	0.25	0.15	0.20	0.25	0.15	0.20
	**Mutation time**	3	3	3	3	3	4	4	4	2	2	2	2
	**Crossover rate**	1.0	0.8	0.9	1.0	1.0	1.0	1.0	1.0	1.0	1.0	1.0	1.0
60 feature results	**s1 (boot=1000)**	0.7681	0.8845	0.8774	0.8780	0.8840	0.8793	0.8849	0.8819	0.8850	0.8770	0.8879	0.8932
	**s2 (boot=1000)**	0.7935	1.0078	0.9952	1.0048	1.0104	0.9922	1.0321	1.0197	1.0035	0.9992	1.0369	1.0247
	**s3 (boot=1000)**	0.7647	0.6023	0.6119	0.5983	0.6070	0.6064	0.6019	0.6098	0.6089	0.6031	0.6018	0.6035
40	**s1**	0.8281	0.8091	0.8065	0.8015	0.7995	0.8090	0.8098	0.8131	0.8097	0.8173	0.8090	0.8017

feature results	**(boot=1000)**												
	**s2 (boot=1000)**	0.9805	0.9024	0.9001	0.9055	0.9009	0.8829	0.9219	0.9069	0.9107	0.9137	0.9344	0.8938
	**s3 (boot=1000)**	0.6578	0.6466	0.6376	0.6362	0.6413	0.6398	0.6519	0.6588	0.6559	0.6550	0.6536	0.6463
20 feature results	**s1 (boot=1000)**	0.7681	0.7557	0.7535	0.7722	0.7674	0.7540	0.7659	0.7559	0.7586	0.7521	0.7639	0.7573
	**s2 (boot=1000)**	0.7935	0.8200	0.7927	0.7938	0.8831	0.7725	0.7829	0.7499	0.7696	0.7702	0.8114	0.7587
	**s3 (boot=1000)**	0.7647	0.7615	0.7581	0.7630	0.7609	0.7804	0.7434	0.7655	0.7642	0.7681	0.7676	0.7869

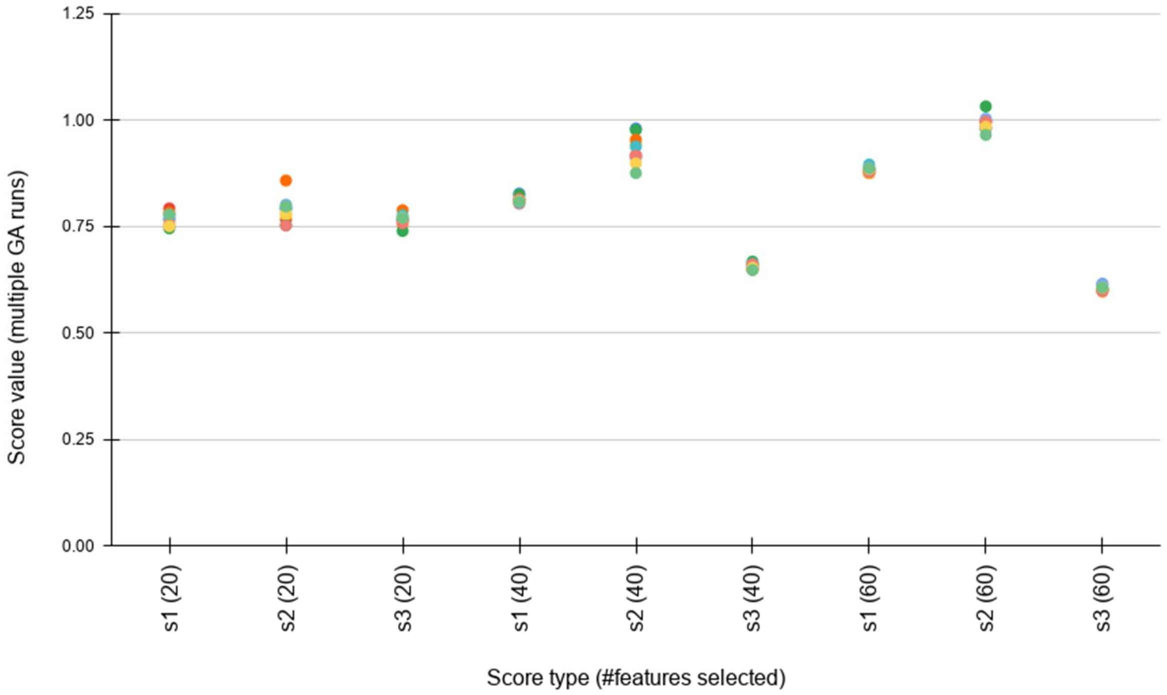

Supplementary Figure S1: Assessment of robustness of GA models under different initialization conditions. A total of 10 runs are attempted for each GA model and performance scores s1, s2 and s3 for each the target feature size is shown as a single point in the plot. Parameter settings for all these runs are as used in the original DREAM submission. Overall variance in most GA runs is less than 5% of the mean value of the score with average variance across all runs being just 2 percentage points, suggesting that the GA initialization does not impact the final model performance and the trained models are highly robust in terms of their predictive performance levels.
